# Perceived Risk of Being Infected With SARS-CoV-2: A Perspective From Indonesia

**DOI:** 10.1017/dmp.2020.351

**Published:** 2020-09-10

**Authors:** Harapan Harapan, Samsul Anwar, Firzan Nainu, Abdul M. Setiawan, Amanda Yufika, Wira Winardi, Alex Kurniawan Gan, Hizir Sofyan, Mudatsir Mudatsir, Rina Suryani Oktari, Abram L. Wagner

**Affiliations:** Medical Research Unit, School of Medicine, Universitas Syiah Kuala, Banda Aceh, Aceh, Indonesia; Tropical Disease Centre, School of Medicine, Universitas Syiah Kuala, Banda Aceh, Aceh, Indonesia; Department of Microbiology, School of Medicine, Universitas Syiah Kuala, Banda Aceh, Aceh, Indonesia; Department of Statistics, Faculty of Mathematics and Natural Sciences, Universitas Syiah Kuala, Banda Aceh, Indonesia; Faculty of Pharmacy, Hasanuddin University, Tamalanrea, Makassar, Indonesia; Department of Microbiology, Faculty of Medicine and Health Sciences, Maulana Malik Ibrahim State Islamic University of Malang, Malang, East Java, Indonesia; Department of Family Medicine, School of Medicine, Universitas Syiah Kuala, Banda Aceh, Aceh, Indonesia; Department of Pulmonology and Respiratory Medicine, School of Medicine, Universitas Syiah Kuala, Banda Aceh, Indonesia; Tsunami & Disaster Mitigation Research Centre (TDMRC), Banda Aceh, Indonesia; Department of Epidemiology, University of Michigan, Ann Arbor, Michigan, Michigan

**Keywords:** COVID-19, perceived risk, preventive measure, SARS-CoV-2, social distancing

## Abstract

**Objectives:**

The aim of this study was to determine the level of coronavirus disease 2019 (COVID-19) risk perceptions in Indonesia and characterize predictors of perceptions.

**Methods:**

An online cross-sectional study was conducted. A questionnaire assessed perceived risk and collected independent variables, including sociodemographic data. A multivariable linear regression model was used to characterize the relationship between independent variables and perceived risk.

**Results:**

We included 1379 respondents in the final analysis with the mean and median of perceived risk score was 19.21% and 10.0%, respectively. Respondents aged between 21 and 30 years had the highest perceived risk, and those who were unmarried had 4.3% higher perceived risk compared with those who were married. Compared with the lowest monthly income group, those making Indonesian Rupiah (IDR) 6-10 million and more than IDR 10 million a month believed they had 4.2% and 8.8% higher risk, respectively. Citizens who lived in cities and health-care workers also had a higher perceived risk compared with those in the rural areas and non–health-care workers, respectively.

**Conclusions:**

Perceived risk of COVID-19 in Indonesia is relatively low, and this could hamper the adoption of preventive measures of COVID-19. Efforts to increase the awareness and perceived risk are important to prevent the pandemic from escalating.

Since the World Health Organization (WHO) declared coronavirus disease 2019 (COVID-19) as a global pandemic on March 11, 2020, more than 45 million cases have been reported^1^ and it has become a major international threat.^2^ All age groups are susceptible to infection, but older age groups have more severe outcomes.^2^ The progression and severity of the disease is associated with dysregulation of the host immune responses.^2^ Indonesia is one of the countries with the highest number of COVID-19 cases in Asia.^1^

Without any specific medical treatment and no vaccine available, social distancing may be one of the only effective measures to reduce virus transmission and to control the disease. People’s compliance with this measure depends on how people act toward risk-seeking or risk-avoiding (ie, public risk perception). A study to assess the perceived risked of COVID-19 has been conducted in the United States and found that perceived risk of infection from COVID-19 was relatively high at 30%,^3^ but there is little evidence about perceived risk in low- and middle-income countries (LMICs) and no study has been conducted in Southeast Asia. People’s perception of risk is influenced by many factors such as knowledge, familiarity, and a feeling of control toward the threat.^4^ We undertook a study to assess the level of perceived risk of COVID-19 and its associated factors in the general population in Indonesia. The results provide important insights in adjusting and enhancing social distancing programs in the region.

## METHODS

### Study Design and Study Instrument

A cross-sectional study was conducted between March 25 and April 6, 2020, to assess the perceived risk of COVID-19 among community members in Indonesia. At the time of this study, there was a recommendation in Indonesia for individuals to stay at home.

Due to restrictions in physical research because of the current pandemic, we conducted an online data collection using Google Forms. The questionnaire was distributed through WhatsApp, the most used communication platform in Indonesia. A questionnaire was developed, tested among small group of pretesters, and was revised before actual study. Using the questionnaire, which required approximately 10 min to complete, we assessed the participants’ perceived risk of being infected with COVID-19 and collected some independent variables, such as sociodemographic data and exposure to COVID-19 information. The protocol of this study was approved by the Institutional Review Board of the School of Medicine, Universitas Syiah Kuala, and National Health Research and Development Ethics Commission of Indonesian Ministry of Health.

### Measures

The outcome in this study was perceived risk of COVID-19. It was defined as the perceived risk of being infected within the next 1 mo by asking the participants to respond to the question, “*What do you think are the chances that you will get coronavirus in the next month?*” The response was assessed on a scale of 0% to 100%. The question was based on previous studies in the United States.^3^ For the statistical analysis, this outcome was treated as a continuous variable.

Independent variables included sociodemographic characteristics (age, gender, educational attainment, occupation, religion, marital status, individual monthly income, and type of urbanicity) and exposure to COVID-19 information (whether or not they have heard about COVID-19 before the survey). For statistical analysis, participants were classified into 5 age groups and 3 levels of educational attainment: junior/senior school graduates, diploma graduates, and university graduates/postgraduates. Based on occupation, participants were divided into civil servants, private sector employees, entrepreneurs, students, or retired, as explained previously, whereas individual monthly income was categorized into 4 groups: less than 2.5 million Indonesian Rupiah (IDR), 2.5-5 million, 6-10 million, and more than 10 million, equal to <US$ 154.7, US$ 154.7-US$ 309.4, US$ 371.2-$ 618.8 and >US$ 618.8, respectively, using an April 2020 exchange rate.

### Statistical Analysis

To assess the relationship between independent variables and perceived risk, a multivariable linear regression model was used. The normality of the residual distribution, homoscedasticity, and linearity were assessed prior analyzing the data. The analysis was conducted in 2 steps. In the initial model, all independent variables were included in the model and all of variables that had *P* < 0.05 in this model were then included in the final model. For both models, β estimates indicate the percentage point difference between the categories compared with a predefined reference category (R). To assess the precision of the estimates, 95% confidence intervals (CI) were calculated. All analyses were performed using SPSS software (SPSS Inc., Chicago, IL).

### Ethics Statement

The protocol of this study was approved by Institutional Review Board of the School of Medicine, Universitas Syiah Kuala, and National Health Research and Development Ethics Commission of Indonesian Ministry of Health.

## RESULTS

### Demographic Characteristics

During the study period, 1402 responses were received, and 23 of them were excluded due to incomplete data, leaving 1379 (98.3%) responses in the data analysis. The mean of respondents’ age was 29.0 y (ranged between 17 and 70 y old) and half (51.4%) aged between 21 and 30 y old (Table 1). The majority (65.7%) of the respondents were female. None of respondents had an educational attainment as primary school or lower and at least, 66.1% of participants were graduated from a university. Almost half earned less than IDR 2.5 million in a month (equal to US$ 154.7), and 76.6% lived in urban areas. There were 264 (19.4%) participants who self-identified as health-care workers (HWCs).

**TABLE 1:** Initial and Final Model of Linear Regression Analyses Showing Factors Associated With Perceived Risk to Be Infected With SARS-CoV-2 in Indonesia (*n* = 1,379)

Variable	*n* (%)	Mean Perceived Risk Score (±SD)	Initial Model		Final Model	
			ß (95% CI)	*P*–Value	ß (95% CI)	*P*–Value
Age group (y)						
<20 *(Reference, R)*	201 (14.8)	15.0% (18.6%)	1		1	
21-30	698 (51.4)	21.2% (22.3%)	4.2% (0.1%, 8.4%)	0.045	4.7% (1.1%, 8.2%)	0.010
31-40	312 (23.0)	18.5% (22.1%)	2.3% (−3.2%, 7.9%)	0.408	2.5% (−2.4%, 7.4%)	0.324
41-50	77 (5.7)	17.3% (22.5%)	1.5% (−5.5%, 8.5%)	0.670	1.8% (−4.7%, 8.3%)	0.590
>51	71 (5.2)	16.6% (24.2%)	−3.1% (−10.7%, 4.5%)	0.422	−1.1% (−8.0%, 5.7%)	0.746
Gender						
Male *(R)*	466 (34.3)	19.9% (22.4%)	1			
Female	893 (65.7)	18.9% (21.7%)	−1.5% (−4.0%, 0.9%)	0.221		
Educational attainment						
Junior/senior school graduated	379 (27.9)	15.9% (19.8%)	1			
Diploma graduated	82 (6.0)	20.2% (25.0%)	2.7% (−3.0%, 8.3%)	0.357		
University graduated/post-graduated	898 (66.1)	20.5% (22.4%)	2.0% (−1.5%, 5.4%)	0.267		
Occupation						
Civil servant *(R)*	270 (19.9)	19.9% (23.1%)	1			
Private sector employee	375 (27.6)	21.2% (23.6%)	−0.4% (−4.1%, 3.3%)	0.825		
Entrepreneur	186 (13.7)	16.6% (21.2%)	−1.5% (−6.0%, 2.9%)	0.505		
Student	511 (37.6)	18.2% (19.9%)	1.5% (−3.7%, 6.6%)	0.580		
Retired	17 (1.3)	25.6% (28.5%)	10.3% (−0.5%, 21.1%)	0.063		
Religion						
Islam *(R)*	1229 (90.4)	19.0% (22.1%)	1			
Buddhism	41 (3.0)	24.1% (19.5%)	3.3% (−3.5%, 10.1%)	0.336		
Christian	52 (3.8)	17.5% (20.9%)	−2.6% (−8.6%, 3.5%)	0.407		
Catholic	28 (2.1)	22.5% (21.5%)	2.7% (−5.5%, 10.9%)	0.525		
Others	9 (0.7)	22.2% (24.4%)	2.3% (−11.9%, 16.5%)	0.751		
Marital status						
Single *(R)*	760 (55.9)	20.0% (21.2%)	1		1	
Married	599 (44.1)	18.2% (22.8%)	−3.9% (−0.3%, −7.4%)	0.032	−4.3% (−1.0%, −7.7%)	0.011
Monthly income (Indonesian Rupiah)						
<2.5 million *(R)*	645 (47.5)	17.6% (20.3%)	1		1	
2.5-5 million	422 (31.1)	19.7% (23.3%)	1.5% (−1.8%, 4.8%)	0.378	1.6% (−1.4%, 4.6%)	0.292
6-10 million	195 (14.3)	20.7% (22.6%)	4.1% (−0.2%, 8.4%)	0.064	4.2% (0.4%, 8.1%)	0.032
>10 million	97 (7.1)	25.2% (24.1%)	8.8% (3.4%, 14.3%)	0.001	8.8% (3.7%, 13.8%)	0.001
Urbanicity						
Rural *(R)*	318 (23.4)	16.2% (21.6%)	1		1	
Urban	1041 (76.6)	20.1% (22.0%)	3.2% (0.4%, 6.0%)	0.025	3.6% (0.9%, 6.4%)	0.009
Health-care worker						
No *(R)*	1095 (80.6)	17.7% (20.9%)	1		1	
Yes	264 (19.4)	25.4% (25.0%)	7.9% (4.7%, 11.2%)	<0.001	7.8% (4.8%, 10.8%)	<0.001
Have heard about COVID-19						
No *(R)*	23 (1.7)	10.4% (16.4%)	1			
Yes	1336 (98.3)	19.4% (22.0%)	5.7% (−3.3%, 14.7%)	0.212		
R^2^			0.056		0.048	
F (*P*-value)			3.690 (<0.001)		6.919 (<0.001)	
MSE			0.046		0.046	

Abbreviation: MSE, mean squared error.

### Perceived Risk of COVID-19 and Its Associated Variables

The mean and median perceived risk of COVID-19 were 19.21% and 10.0%, respectively. In the final model, being 20-30 y old (compared with those less than 20 y old), being unmarried, having a higher individual monthly income, living in the urban cities, and being a HCW were associated with higher perceived risk (Table 1). Respondents between 21 and 30 y old had approximately 4.7% higher perceived risk compared with those who aged less than 20 y old (95% confidence interval [CI]: 1.1%, 8.2%). The perceived risk of infection was 4.3% lower among those who were married than unmarried respondents. Those who earned IDR 6-10 million or more than IDR 10 million a month believed they had 4.2% (95% CI: 0.4%, 8.1%) and 8.8% (95% CI: 3.7%, 13.8%) higher risk compared with those from the least wealthy group, respectively. Those who lived in cities had 3.6% higher perceived risk compared with respondents who lived in rural areas (95% CI: 0.9%, 6.4%), and HCWs also believed they had higher perceived risk being infected compared with non-HCWs (7.8%, 95% CI: 4.8%, 10.8%).

## DISCUSSION

We set out in this study to assess the risk perception of Indonesian citizens toward COVID-19. This is an important issue to consider because successful implementation of preventive measures against COVID-19 relies on public perceptions of risk,^5^ along with self-efficacy of the particular action. We found that perceived risk of COVID-19 in Indonesia was 19.21%, which is lower than a study in the United States (32.0%).^3^

Several demographic groups had higher risk perceptions. We identified that those between 20 and 30 y old had the highest perceived risk, followed by those 31-40 y old, and this finding is similar those of a study in the United States that found millennials (aged 24-39 y old) had higher perceived risk than those 56 y old or older.^3^ One possible reason is that younger adults are more likely to work in public settings. In addition, our study found that being unmarried was associated with higher perceived risk of getting infected with COVID-19 compared with those who were married. According to the Indonesian population structure, people who are unmarried are more likely to be younger. Overall, individuals with single status and younger age may be more exposed to information about the current pandemic through social and mass media,^6^ which might increase their anxiety and perceived risk.

Our data indicate that level of income is monotonically related to increased perceptions of the risk of severe acute respiratory syndrome coronavirus 2 (SARS-CoV-2) infection. However, a study in the United States found that individuals with lower income had higher perceived risk of influenza infection, possibly resulting from increased emotional reactions to the imbalanced distribution of health services.^7^ Another possible explanation for the discrepancy in the trend of risk perception may be due to the availability of effective treatments. In the case of Middle East respiratory syndrome (MERS) and COVID-19, there is not yet an effective therapeutic agent or vaccine,^2^ so people may have fewer concerns on the fair provision of health services. For influenza, there are widely available vaccines and pharmaceutical interventions. The availability of effective treatments to manage infectious diseases may have less impact on risk perception of wealthy individuals because they have a greater access to health services than their lower-income counterparts. While currently available data are limited to conclude the relationship of social status with perceived risk of individuals across different infectious diseases, it is important to note that our result provides a valuable insight in the importance of individuals’ financial capacity to respond to infectious disease threat and, perhaps, to comply to public health preventive measures. As an effective treatment or vaccine becomes available, not only may individuals’ risk perception change, but this may change differentially based on the individual’s financial background.

Increased perceptions of being infected with COVID-19 was also predominantly among people who lived in the urban areas. We speculated that this is probably due to increased awareness of individuals in cities and greater difficulties to maintain social distancing in urban settings. Mass gatherings have been shown to correlate with increasing risk of SARS-CoV-2 transmission, and mass gatherings are more likely to happen in urban areas, such as in offices, shopping centers, public transportation, and leisure facilities. As a result, city dwellers may have increased alertness (and anxiety) about SARS-CoV-2 transmission. Future research could more clearly examine the reason behind this relationship between urbanicity and risk perceptions by measuring social distancing practices of respondents.

HCWs act as the front-liner workers during the pandemic, and this might explain why the perceived risk of getting infected with SARS-CoV-2 was higher among HCWs compared with non-HCWs in this present study. Another factor that might influence perceived risk among HCWs is the availability of infection control systems at their work places, such as protective gear and supportive facilities.^8^ As this study was conducted at the beginning of COVID-19 pandemic in Indonesia where most of health-care facilities were not prepared with infection control yet, it might explain why HCWs had higher perceived risk of being infected.

Perceived risk is associated with disaster preparedness in communities,^9,10^ including during the pandemic. Risk perception predictive of engagement in protective behaviors, such as the use of personal protective gear, maintaining social distancing, and eventually obtaining a vaccine. Public trust plays an important role in shaping the willingness of the people to comply with public health authorities’ recommendations,^5,10^ which in turn will lead to the successful implementation of non-medical intervention during COVID-19 pandemic. To gain public trust, the government must provide comprehensive information about COVID-19 and provide basic needs for its people. Failure in doing so may lead to negative impairment on socio-economic activities and the emergence of public distrust. Future studies are warranted to confirm the role of social status, educational background, occupation, and/or living environment to the level of perceived risk displayed by individuals and whether this notion is true across different settings.

There are some limitations of the study. We did not measure social distancing practices; therefore, this study is unable to elucidate whether higher perceived risk resulted from respondents being unable to adopt social distancing practices (eg, because of work), or if it was because they were more knowledgeable or aware of COVID-19. Previous research has identified selection biases arising from Internet samples because the survey can be completed only by those who have access to the Internet and the Internet infrastructure varies by region in Indonesia. However, Internet-based collection is the most feasible approach during the current pandemic.

## CONCLUSIONS

Perceived risk of COVID-19 in Indonesia is relatively low, with the mean value of approximately 19.21%. High perceived risk is associated with being a young adult, being single, having a high monthly income, living in the cities, and being a HCW. Health and risk communications should come from trusted and reliable programs and can be used to enhance risk perceptions in the future. Higher risk perceptions may correlate with increased compliance with rules and recommendations released by the government and public health authorities amid the current COVID-19 pandemic.
